# Pharmacological rationale for antihypertensive drug choice on COVID‐19–affected patients: ACEI/ARB might not increase their susceptibility

**DOI:** 10.1111/jcmm.15850

**Published:** 2020-09-17

**Authors:** Hong‐Jin Zhao, Xiao‐Mei Yang, Ai‐Hong Wang, Yan Li

**Affiliations:** ^1^ Department of Cardiology Shandong Provincial Hospital affiliated to Shandong First Medical University Ji’nan China; ^2^ Department of Anesthesiology Qilu Hospital Cheeloo College of Medicine Shandong University Ji’nan China; ^3^ School of Medicine Cheeloo College of Medicine Shandong University Ji’nan China

Dear Editor,

The novel coronavirus outbreak is threatening human health globally mainly through causing severe acute respiratory syndrome (SARS), and this coronavirus is named as SARS‐CoV‐2 (severe acute respiratory coronavirus 2) to be differentiated from SARS‐CoV in 2003. SARS‐CoV‐2 spreads mainly through the respiratory route and uses membrane‐bound ACE2 (angiotensin‐converting enzyme 2) as the receptor to enter into the cells for replication.^1^ The receptor ACE2 is a single‐pass membrane protein, and its extracellular enzymatic domain is classically known to convert Ang II (angiotensin II) to Ang1‐7 in RAS (renin‐angiotensin systems).^2^ When cleaved from the cell membrane, ACE2 enters into the blood and this secreted ACE2 loses the ability to mediate the coronavirus infection. The membrane‐bound ACE2‐mediated SARS infection consumes a large proportion of ACE2 molecules, leading to the down‐regulation of ACE2/Ang1‐7 and up‐regulation of Ang II. This imbalance of the RAS system eventually results in exacerbation of inflammation in lung tissues and cardiovascular dysfunction.^3^ To avoid the adverse effects of Ang II on the cardiovascular system, ACEI/ARB (angiotensin‐converting enzyme inhibitors/Ang II receptor blockers) is used as the first‐line medicine in the treatment of hypertension and heart failure through inhibiting the production or function of Ang II.^4^ Considering the large consumption of ACE2 due to the SARS infection and the subsequent increased Ang II accumulation, the drug choice ACEI/ARB should be more suitable for the treatment of hypertensive COVID‐19 (coronavirus disease 2019) patients. This drug strategy will rebalance the RAS system and prevent the progression from pneumonia to severe ARDS in theory.

Recently, Fang et al^5^ have suggested the conversion of ACEI/ARB to CCB (calcium channel blockers) for hypertensive patients based on studies reporting the up‐regulatory effects of ACEI/ARB on ACE2 expression, which could contribute to increased sensitivity to SARS‐CoV‐2 infection. A study has shown the increased cardiac ACE2 mRNA levels by ACEI/ARB administration.^6^ Moreover, ARB has been implicated in up‐regulating the cardiac and renal ACE2 protein levels.^7^, ^8^ However, Western blot data are lacking to experimentally distinguish ACE2 molecules in full‐length membrane form from those in cleaved/secreted form due to their similar molecular weight. As a result, a note of caution is needed when interpreting the roles of ACE2 expression levels in SARS‐CoV‐2 infection as the cleaved ACE2 could not function as the receptor for coronavirus to invade the target cells. Importantly, the existing evidence does not support the exclusion of ACEI/ARB from the treatment of hypertensive patients without/with COVID‐19.

Hilliard et al^9^ have demonstrated the up‐regulation of ACE2/Ang1‐7 pathways in pre‐menopausal women, compared with older women, suggesting the lower expression and/or activity of ACE2 in ageing women. Similarly, Xie et al^10^ have displayed that the expression of ACE2 in rat lung dramatically declines as ageing in both male and female rats. However, the ratio of severe COVID‐19 cases rises along with ageing, with the highest ratio in ≥65‐year‐old patients (44/153 = 28.7%)^11^ which is reversely correlated to the lung ACE2 levels. Moreover, SARS‐CoV in 2003, sharing the same receptor ACE2, shows different age group preferences in contrast to SARS‐CoV‐2. Furthermore, SARS‐CoV was prone to attack aged males (≥75‐year‐old) compared with aged females,^12^ though aged females exhibit significantly higher lung ACE2 levels.^10^ These data indicate that the ACE2 expression levels in the lung per se cannot determine the susceptibility or the severity of coronavirus diseases.

However, there is one pre‐print geospatial study showing ARB might be linked to increased COVID‐19 morbidity using the physician's prescription information in 2017.^13^ Of note, this conclusion is less convincing because the prescription of ARB in 2017 did not mean the continuation of ARB application during the outbreak of COVID‐19 in 2020. In contrast, with the latest medication data before COVID‐19 diagnosis, Reynolds et al have recently reported neither ACEI nor ARB application is associated with increased COVID‐19 incidence through case‐control study.^14^ In addition, according to papers Fang et al cited,^11^, ^15^ the comorbidity rate of hypertension in COVID‐19 (15%‐30%) is comparable or even lower than the hypertension morbidity in Chinese population (27.8%),^16^ further implicating SARS‐CoV‐2 is not prone to infect hypertensive patients though many of them use ACEI/ARB as hypertensive treatment. Notably, the comorbidity of hypertension in severe COVID‐19 patients is much higher.^9^, ^11^ However, ACEI/ARB has little relationship with these severe COVID‐19 patients because ACEI/ARB was changed to other antihypertensive treatment at that time once COVID‐19 was confirmed according to the initial guidelines in China, and the discontinuation of ACEI/ARB usually occurred at least one week before COVID‐19 patients progressed to the severe cases. On the contrary, these data might hint the disadvantages of ACEI/ARB withdrawal from the treatment of hypertensive COVID‐19 patients. In consistence, recent clinical research has implicated that the application of ACEI/ARB in hospitalized COVID‐19 patients with hypertension has a lower risk of all‐cause mortality in contrast to ACEI/ARB non‐users.^17^

Currently, it is generally believed that SARS‐CoV‐2 infection exhausts a great number of ACE2 molecules, which leads to decreased ACE2 expression and increased angiotensin II levels, resulting in RAS imbalance. Such a RAS imbalance has been widely accepted to account for the exacerbation of inflammatory pneumonia and hypertension. Therefore, to achieve RAS system rebalance, most Chinese experts now recommend the administration of ACEI/ARB for the hypertensive treatment, especially in COVID‐19 patients.

## CONFLICT OF INTEREST

The authors confirm that there are no conflicts of interest.

## AUTHOR CONTRIBUTION

**Hong‐Jin Zhao:** Supervision (equal); Writing‐original draft (equal); Writing‐review & editing (equal). **Xiao‐Mei Yang:** Supervision (equal); Writing‐original draft (equal); Writing‐review & editing (equal). **Ai‐Hong Wang:** Supervision (equal); Validation (supporting); Writing‐review & editing (lead). **Yan Li:** Conceptualization (equal); Project administration (equal); Supervision (equal); Writing‐review & editing (equal).

### DATA AVAILABILITY STATEMENT

The authors confirm that citations for available data have been included in References section.
